# Plant Frataxin in Metal Metabolism

**DOI:** 10.3389/fpls.2018.01706

**Published:** 2018-11-21

**Authors:** Diego F. Gomez-Casati, Maria V. Busi, Maria A. Pagani

**Affiliations:** Centro de Estudios Fotosintéticos y Bioquímicos (CEFOBI-CONICET), Universidad Nacional de Rosario, Rosario, Argentina

**Keywords:** frataxin, iron, copper, Fe-S clusters, metal homeostasis

## Abstract

Frataxin is a highly conserved protein from prokaryotes to eukaryotes. Several functions related to iron metabolism have been postulated for this protein, including Fe-S cluster and heme synthesis, response to oxidative damage and oxidative phosphorylation. In plants, the presence of one or two isoforms of this protein with dual localization in mitochondria and chloroplasts has been reported. Frataxin deficiency affects iron metabolism in both organelles, leading to an impairment of mitochondrial respiration, and chlorophyll and photosynthetic electron transport deficiency in chloroplasts. In addition, plant frataxins can react with Cu^2+^ ions and dimerize, which causes the reduction of free Cu ions. This could provide an additional defense mechanism against the oxidation of Fe-S groups by Cu ions. While there is a consensus on the involvement of frataxin in iron homeostasis in most organisms, the interaction of plant frataxins with Cu ions, the presence of different isoforms, and/or the localization in two plant organelles suggest that this protein might have additional functions in vegetal tissues.

## Iron Functions in Plants, Uptake and Distribution

Iron is an essential element for almost all life forms. It is part of cofactors that carry out electron transfer functions, and is involved in chemical transitions (e.g., hydroxylations), hydration and dehydration reactions and radical-mediated rearrangements. Iron also participates in oxygen sensing and transport, and regulation of protein stability (Connorton et al., 2017). Iron is essential for plant growth but, at the same time, is highly reactive and toxic via the Fenton reaction. Thus, plants tightly control iron homeostasis and react to both deficiency and overload of iron. Photosynthetic organisms are distinguished by a high iron requirement for the function of both mitochondria and chloroplasts. These organelles are thought to play a major role in the iron metabolism of the plant cell because this metal/ion serves as an essential cofactor for many enzymes involved in the mitochondrial respiratory chain and electron transfer in the chloroplastic photosynthetic complexes (Morrissey and Guerinot, 2009).

Plants mainly acquire iron from the rhizosphere. Although iron is one of the most abundant metals in the land surface, its availability for plant roots is very low, dependent on the soil reduction potential and pH. In soils that are aerobic or at higher pH, Fe is readily oxidized and is thus predominately in the form of insoluble ferric Fe(III) oxides. At lower pH, the ferric ion is freed from the oxide and becomes more available for uptake. Thirty percent of the world’s land for cultivation is too alkaline for optimal plant growth – the most common problem is iron deficiency. Many plant foods like rice, maize, and wheat constitute poor sources of dietary iron (Takahashi et al., 2001; Walker and Connolly, 2008; Morrissey and Guerinot, 2009).

Iron uptake in plants has classically been divided into Strategy I (reducing, dicotyledonous and non-graminaceous monocots) and Strategy II (chelating, graminaceous monocots), the main difference being the oxidation state of the iron (Romheld and Marschner, 1986). In the rhizosphere, iron is mostly found as ferric oxyhydrates of very low solubility. The participation of FRO2 (Ferric Reduction Oxidase 2), IRT1 (Iron-Regulated Transporter 1) and AHA2 (a proton pump located in plasma membrane) has been reported in Strategy I in tomato and *Arabidopsis thaliana*. Phytosiderophores (plant-derived small organic molecules with a high affinity for iron) and oligopeptide transporters participate in Strategy II in rice, maize and barley (including the iron-siderophore transporters YS1, first characterized in maize, and YSL15 in rice) (Higuchi et al., 1999; Curie et al., 2001; Inoue et al., 2009; Nozoye et al., 2011). Rice is particular among monocots because it uses Strategy II to acquire Fe from rhizosphere but also has the Strategy I-like system (Ishimaru et al., 2006). Under flooded conditions, when Fe(II) is more stable and abundant, rice also absorbs Fe(II) directly via OsIRT1 (Oryza sativa IRT1) and OsNRAMPs (Natural Resistance Associated Macrophage Proteins) (Takahashi et al., 2011). It has been reported that IRT1 has a major role in the regulation of plant iron homeostasis and it is essential for plant growth under iron-limited conditions (Vert et al., 2002). IRT1 undergoes ubiquitin-dependent endocytosis to prevent the uptake of other divalent metals such as Mn, Zn, and Co (Barberon et al., 2014; Dubeaux et al., 2015). In addition, other transporters such as several NRAMPs also participate in the uptake of iron, but as a low-affinity systems (Curie et al., 2000; Castaings et al., 2016).

Several phenylpropanoid-pathway enzymes are upregulated under iron deficiency in *Arabidopsis* such as PAL1, PAL2 (two Phe ammonia-lyases), 4CL1 and 4CL2 (two 4-coumarate:CoA ligases), the ABC transporter PDR9 (responsible for coumarin secretion into the rhizosphere) and MAT3 (an enzyme that produces *S*-adenosyl methionine which is involved in coumarin biosynthesis) (Lan et al., 2011; Rodriguez-Celma et al., 2013; Mai et al., 2016). It has been demonstrated that the phenolic compounds secreted facilitate Fe(III) availability for the FRO2 reductase to generate Fe(II) which is transported by IRT1 (Fourcroy et al., 2016). This suggests that these phenolic compounds are important in/for Strategy I iron uptake and are also involved in iron mobilization from insoluble pools (soil) or root apoplast (Fourcroy et al., 2014; Schmid et al., 2014). The catecholic coumarins found at the highest levels in the exudates of iron-deficient wild-type *Arabidops*is are esculetin, fraxetin, scopoletin, and sideretin (Fourcroy et al., 2014; Schmid et al., 2014). Fraxetin is the major coumarin exuded into the rhizosphere in response to iron deficiency in alkaline conditions, while sideretin is exuded in acidic conditions. Fraxetin and sideretin are synthesized from scopoletin by hydroxylases [2-ODD (S8H)], which generates fraxetin, and a cytochrome P450 (CYP82C4), which oxidizes fraxetin to generate sideretin) and both compounds efficiently mobilize and reduce insoluble Fe(III), rescuing the chlorotic phenotypes (Rajniak et al., 2018). To date, most efforts in understanding soil iron-uptake limitations have focused on the role of soil pH and have ignored other potentially relevant factors such as interactions with soil organic matter or other metals such as Zn(II) or Mn(II) (Rajniak et al., 2018).

Because of its low solubility and high toxicity, iron must form complexes with chelators to be translocated without causing damage by redox reactions (Dubeaux et al., 2015). One of the complexes necessary for transport by the symplast occurs between the reduced form of iron (Fe^2+^) and nicotianamine, a non-protein amino acid produced by nicotianamine synthase (Inoue et al., 2003; Klatte et al., 2009; Rellan-Alvarez et al., 2010), which also chelates other divalent cations (e.g., Zn^2+^). Once iron has passed the endodermis, it is taken into the xylem to reach the shoot. The dominant form of iron in the xylem is Fe^3+^ bound to citrate and, therefore, the flow of citrate is essential for iron translocation (Rellan-Alvarez et al., 2010). This translocation is mediated by the efflux transporter FRD3 in *Arabidopsis* (Green and Rogers, 2004) and its ortholog FRDL1 in rice (Yokosho et al., 2016).

The leaves are the main receptor organ for iron because they are where it is needed for photosynthesis. Here, iron re-enters the symplast by the action of FRO proteins (Finazzi et al., 2015; Bashir et al., 2016). Subsequently, it can be remobilized and arrives at other sink organs through the phloem, where it is mainly transported as Fe(II)- nicotianamine complexes. In *Arabidopsis*, OPT3 (of the oligopeptide transporter family protein) is involved in this process (Mendoza-Cozatl et al., 2014; Zhai et al., 2014). The seed is considered the final destination of iron, where iron reserves are fundamental for germination. The YSL transporters are involved in the iron loading of the seeds (Le Jean et al., 2005).

Mitochondria and chloroplasts represent major iron sinks within cells, as iron is required for the proper functioning of the respiratory chain and photosynthetic protein complexes. The mechanisms by which Fe is obtained by chloroplasts and mitochondria are not as well-known as iron uptake at the root epidermis. There is evidence of reduction-based mechanisms for chloroplast and mitochondria Fe-acquisition, since the presence of FRO7 and FRO3/FRO8 has been reported in each organelle (Jeong et al., 2008). A mitochondrial iron transporter, which belongs to the mitochondrial carrier family (MCF) also present in yeast, zebrafish, humans, and *Drosophila*, was identified in rice and named MIT (Bashir et al., 2011). The protein PIC1 (Permease In Chloroplasts 1) was the first molecular component involved in plastid Fe-transport identified in *Arabidopsis* and tobacco (Duy et al., 2007; Gong et al., 2015). In addition, other proteins are supposed to participate in Fe transport across the chloroplast envelope, including two transporters from the yellow stripe 1-like family, YSL4 and YSL6, which have been characterized as potential plastid Fe-efflux transporters in *Arabidopsis* (Divol et al., 2013). However, in summary, mitochondrial and chloroplast iron transport is till far from being completely deciphered, nor it is understood their cross talk regarding iron homeostasis.

## Biosynthesis of Iron Cofactors: the Participation of Frataxin

Iron is an important component of Fe-S clusters found in many ferrosulfoproteins. Three different systems capable of mediating Fe-S cluster assembly have been identified: NIF (nitrogen fixation) found in azeotropic bacteria, SUF (mobilization of sulfur) found in archaea, bacteria and plastids, and ISC (iron-sulfur cluster) present in bacteria and mitochondria (Romheld and Marschner, 1983; Patzer and Hantke, 1999; Dos Santos et al., 2004; Lill and Muhlenhoff, 2008; Morrissey and Guerinot, 2009; Balk and Pilon, 2011). These systems have in common three steps: (i) the production of sulfur from cysteine, catalyzed by a cysteine desulfurase; (ii) the assembly of Fe-S clusters onto scaffold proteins; and (iii) the transfer of the mature Fe-S cluster into an apoprotein. It has been reported that a small mitochondrial protein, frataxin, could be involved in several steps of this process (Balk and Lobreaux, 2005; Lill and Muhlenhoff, 2008; Lillig and Lill, 2009; Balk and Pilon, 2011; Turowski et al., 2012).

Frataxin is a nuclear-encoded mitochondrial protein whose deficiency is the cause of Friedreich’s ataxia, a hereditary cardio- and neurodegenerative disease in humans. This protein plays a role in Fe-S cluster biosynthesis, protection against oxidative stress and iron metabolism. Frataxin is highly conserved throughout evolution, being present in humans, plants, flies, worms, and bacteria (Gibson et al., 1996; Babcock et al., 1997; Adinolfi et al., 2002; Busi and Gomez-Casati, 2012; Han et al., 2017). Some hints about frataxin function can be gleaned from the evolutionary record (Morrissey and Guerinot, 2009). The appearance of frataxin in eukaryotes occurred about the time of the endosymbiotic event creating mitochondria from the purple bacterial ancestor, and it was probably acquired by mitochondria together with other components of the *ISC* operon (Waters et al., 2007; Morrissey and Guerinot, 2009). Different organisms developed mechanisms to avoid the toxicity of free metal ions that allow the control of their uptake, storage and release. It was postulated that frataxin potentially fulfills some of these functions (Busi and Gomez-Casati, 2012).

**FIGURE 1:**
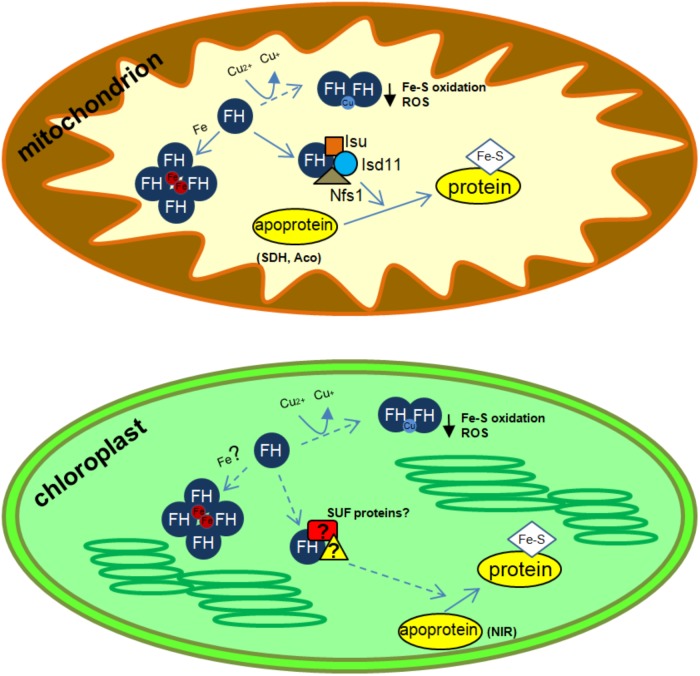
Schematics of possible frataxin functions in plant organelles. ROS, reactive oxygen species; SDH, succinate dehydrogenase; Aco, aconitase; NIR, nitrite reductase. In mitochondria, frataxin increase Nfs1 activity and could also be involved in several additional functions related to iron and copper metabolism (iron storage, Fe-S protein maturation and prevention of ROS formation after Cu reduction). In chloroplasts, frataxin could be involved in metal metabolism and Fe-S synthesis, possibly by the interaction with proteins from the SUF system. In addition, frataxin could also be involved in Cu reduction and response to Fe-S cluster oxidation and in the prevention of ROS accumulation. Known frataxin functions are indicated with solid arrows, whereas possible functions are indicated with dashed arrows.

In 2004, we identified the first frataxin protein in a photosynthetic organism, *A. thaliana* (Busi et al., 2004, 2006). The functionality of AtFH was assessed by complementation of a yeast frataxin-null mutant, suggesting that AtFH was involved in plant mitochondrial respiration and stress responses (Busi et al., 2004). In agreement with this hypothesis, AtFH-deficient plants presented retarded growth, showed an increment of reactive oxygen species (ROS) and an induction of oxidative stress markers. Interestingly, we also found an increment of aconitase and succinate dehydrogenase-2 (SDH2-1) transcripts, both coding for mitochondrial Fe-S-containing proteins. The reduction of the activities of both enzymes indicates that AtFH also participates in Fe-S cluster assembly or the insertion of Fe-S clusters into apoproteins, possibly in cooperation with other proteins such as Nfs1, HscB, Isd11, and Isu (among others), by the formation of a multiprotein complex (Gonzalez-Cabo et al., 2005; Busi et al., 2006; Maliandi et al., 2007; Shan et al., 2007; Shan and Cortopassi, 2012; Turowski et al., 2012; Leaden et al., 2014; Figure 1). Consistent with the essential role of AtFH in cellular function is the observation that homozygous null mutants result in a lethal phenotype (Busi et al., 2004, 2006; Vazzola et al., 2007; Martin et al., 2009). Our data also substantiate the hypothesis that AtFH, apart from its role in protecting bioavailable iron within mitochondria and the biogenesis of Fe-S groups, plays a role in the biosynthesis of heme in plants (Maliandi et al., 2011). We have shown, by *in vitro* experiments, that AtFH catalyzes the formation of heme when it is incubated with Fe(II) and protoporphyrin IX (Armas et al., 2019). Despite plant ferrochelatases are only present in chloroplasts (Lister et al., 2001; Masuda et al., 2003), a modest but detectable ferrochelatase activity has been observed in plant mitochondria (Cornah et al., 2002), pointing toward frataxin as the enzyme responsible for the reaction in this organelle.

We have reported that in *Arabidopsis*, AtFH is dual-targeted to mitochondria and chloroplasts (Turowski et al., 2015) and its deficiency alters the normal functioning of chloroplasts by affecting the levels of Fe, chlorophyll, and Fe-S proteins, suggesting that AtFH plays a role as a modulator of both the mitochondrial ISC and chloroplast SUF systems (Turowski et al., 2015; Figure 1). In AtFH deficient plants, we also found a reduction of about 40% in ferredoxin levels and about 30% in nitrite reductase (a chloroplastic Fe-S-containing protein) activity (Turowski et al., 2015). Thus, it is possible that plant frataxins play similar roles in the two organelles, as iron donors, regulating the activity of Fe-S proteins, and, possibly, modulating the activity of the ISC and SUF systems.

Although we identified only one frataxin gene in *A. thaliana*, other plants could have at least two isoforms (Murgia et al., 2009). Recently, we reported the presence of two functional frataxin isoforms in *Zea mays*, ZmFH-1 and ZmFH-2, located in both, mitochondria and chloroplasts (Buchensky et al., 2017). The biochemical, biophysical and physiological studies showed some differences between the two isoforms in protection against oxidants, aggregation state and expression patterns. ZmFH-1 showed to be more efficient against oxidative damage and it is expressed in a higher extent in almost all tissues respect to ZmFH-2. Furthermore, ZmFH-2 undergoes some conformational changes when exposed to air, possibly due to a C-terminal extension that could give high thermodynamic stability to the protein in comparison to ZmFH-1 (Buchensky et al., 2017; Sanchez et al., 2018). These results suggest that the two proteins play similar but not identical roles in plant cell metabolism.

In some cases, frataxin assembly seems to be a consequence of iron incorporation into the protein. The assembly of yeast frataxin, for instance, seems to be driven by iron oxidation and accumulation by iron core formation, whereas iron core degradation results in protein disassembly (Adamec et al., 2000; Aloria et al., 2004). Human frataxin assembly has been proposed to be a means of detoxifying redox-active iron. However, in the case of human frataxin, iron does not seem to be the main factor for assembly and it was postulated that the assembly is a physiological property of the protein that allows it to perform diverse cellular functions (O’Neill et al., 2005a,b).

## Frataxin and Copper

Cu is a trace element necessary for many different processes in plants. More than 50% of Cu present in plants is found in chloroplasts, which underscores the need for Cu in photosynthesis (Raldugina et al., 2016). The major Cu protein is plastocyanin, which is an essential component of the electron transport chain of photosystem I (Schubert et al., 2002; Kramer and Clemens, 2006). Plant mitochondria also require Cu for the assembly and function of cytochrome *c* oxidase, the terminal enzyme of the respiratory chain, among other Cu proteins (Garcia et al., 2014). Nevertheless, free copper ions are dangerous inside cells since they can directly attack functional sites in proteins or induce ROS production through Fenton and Haver–Weiss reactions (Halliwell, 2006). As a result, many protective mechanisms exist, such as Cu metallochaperones (Robinson and Winge, 2010), low molecular weight thiol ligands such as glutathione, and the mitochondrial anionic compound known as copper ligand (or CuL) described in yeast (Cobine et al., 2004). Still, Cu is toxic when present in excess, probably because these housekeeping defense mechanisms are overwhelmed.

Copper toxicity has classically been associated with ROS production and oxidative damage. However, more recent evidence shows that damage and inhibition of Fe-S metabolism is the more likely initial manifestation of Cu toxicity (Dupont et al., 2011). Dehydratase family enzymes are rapidly inactivated upon exposure of *Escherichia coli* cells to low micromolar copper levels due to the displacement of iron atoms from their solvent-exposed Fe-S clusters (Macomber et al., 2007). Copper stress in *Bacillus subtilis* leads to enhanced expression of Fe-S cluster scaffold (SufU) and many Fe-S proteins, as well as iron and sulfur uptake pathways (Chillappagari et al., 2010). Additional work performed *in vitro* confirms that mammalian ISCA1/2 and GLRX5 Fe-S clusters are destabilized by the presence of Cu(I) (Brancaccio et al., 2017).

*De novo* Fe-S cluster biogenesis in plants occurs in chloroplasts and mitochondria and as we mentioned above, plant frataxins are located in both organelles (Turowski et al., 2015; Buchensky et al., 2017). We have shown that frataxins can react *in vitro* with the free cupric ion to give the cuprous form (Sanchez et al., 2018). In this reaction, the conserved cysteine residue of frataxin is oxidized forming a disulfide bridge with another frataxin unit (Buchensky et al., 2017). Moreover, plant frataxins oxidized and in their dimeric form (AtFH and ZmFH2) can bind free Cu(I) ions (Sanchez et al., 2018). Frataxin oxidation and the resulting dimerization might be necessary to bring together enough ligands for the cuprous ion. Cu(I) binding sites are dominated by amino acids with sulfur ligands like cysteine and methionine, but histidine can also bind Cu(I), although less tightly. The cuprous ion is coordinated by 2, 3, or 4 ligands, and proteins involved in copper resistance tend to have a coordination environment of low affinity and high coordination number (usually 4) (Rubino and Franz, 2012). AtFH and ZmFH-2 contain three histidine and two methionine residues (the cysteine residue is oxidized and unable to bind copper). Among the potential plant frataxin Cu(I) ligands, two histidine residues are relatively close (seven and eight amino acids apart in ZmFH-2 and AtFH, respectively). In this manner, a plant frataxin dimer might fulfill the requirements of four low-affinity ligands for a Cu(I) ion. The reaction observed *in vitro* might reasonably occur *in vivo*, with concomitant reduction of free copper ions, hence protecting the Fe-S clusters in plants. In this way, frataxins could comprise an additional defense for Fe-S clusters in the presence of excess copper (Figure 1).

There is additional evidence from different organisms that frataxin might be involved in copper metabolism. Friedreich’s ataxia patients show altered Cu distribution in the dentate nucleus of the central nervous system (Koeppen et al., 2012), and in the *Drosophila* model of the disease there is a generalized increase in copper content (Soriano et al., 2016), as there is in mitochondria of yeast frataxin-null mutants (Han et al., 2017). Moreover, in frataxin knockdown flies, treatment with copper chelators improved their impaired motor performance without altering the iron accumulation phenotype, implying a direct role of Cu in the pathophysiology of the disease (Soriano et al., 2016).

*Saccharomyces cerevisiae* frataxin-null mutants are more sensitive to Cu than wild-type cells (Foury and Cazzalini, 1997; Han et al., 2017) and in anaerobic growth conditions, in which little oxygen is available for ROS generation, copper accumulation and its toxicity increases (Strain and Culotta, 1996; Outten et al., 2001). Ha-Duong and coworkers have shown that yeast frataxin can bind Cu(II) and Cu(I) ions, with higher affinities than iron (Han et al., 2017). Thus, we might assume that yeast frataxin protects Fe-S clusters from copper toxicity by preventing free Cu ions from interacting with them in the same way we have suggested for plant frataxins. Yeast complementation assays with plant frataxins support this hypothesis. *Arabidopsis* and maize frataxins can completely restore the ability of a *S. cerevisiae* frataxin-null strain to grow in high copper medium (Sanchez et al., 2018), which led us to infer that plant and yeast frataxins might perform similar and conserved molecular functions in copper metabolism.

## Author Contributions

DG-C, MB, and MP collaborated in the writing of the manuscript.

## Conflict of Interest Statement

The authors declare that the research was conducted in the absence of any commercial or financial relationships that could be construed as a potential conflict of interest.
